# Sex Determination in Reptiles: A Review

**DOI:** 10.3390/ani15020168

**Published:** 2025-01-10

**Authors:** Alessandro Vetere, Michele Capasso, Francesco Di Ianni

**Affiliations:** 1 Department of Veterinary Science, University of Parma, 43121 Parma, Italy; francesco.diianni@unipr.it; 2 Department of Veterinary Medicine and Animal Production, University of Naples Federico II, 80138 Napoli, Italy

**Keywords:** reptiles, sex identification, sex determination, endoscopy, cloacoscopy, cystoscopy, celioscopy, reproduction

## Abstract

Reptile biodiversity is rapidly declining, with over 11,733 recognized species across 1226 genera being documented, many of which are endangered. Captive breeding programs play a crucial role in conservation; however, effective management requires accurate sex determination, especially due to the fact that many reptile species exhibit minimal or no sexual dimorphism. When present, sexual dimorphism manifests as differences such as size, coloration, and morphological features influenced by evolutionary pressures and hormones. Various sex determination techniques are employed due to the lack of external sexual characteristics in many species. These methods include probing, hydrostatic eversion, popping, ultrasound, CT, radiography, contrast radiography, endoscopy, and genotypic sex determination. Accurate sex determination is crucial for the success of captive breeding programs and the conservation of reptile species. Advanced imaging and molecular methods offer promising non-invasive alternatives but may not be universally accessible or effective. An understanding of the unique reproductive anatomy and the use of appropriate sexing techniques are fundamental to establishing breeding groups, preventing aggressive behaviours among groups, and ensuring the long-term survival of endangered reptile populations.

## Simple Summary

Many reptile species lack obvious physical differences between males and females, thus making sex identification a challenging process. This literature review comprehensively includes all of the current methodologies for sex determination in reptiles, which are updated to today. By thoroughly understanding these updated techniques and the unique reproductive anatomy of reptiles, we can improve breeding programs, minimize aggressive behaviours among individuals, and significantly contribute to the conservation of endangered reptile species. A wide range of techniques, such as probing, manual eversion, imaging methods including ultrasound and computed tomography (CT) scans, endoscopy, and genetic testing, are discussed in this review.

## 1. Introduction

As of March 2022, 1226 genera of reptiles have been documented to exist, with 11,733 currently recognized species [1], most of which are currently endangered or threatened with extinction [2], as biodiversity is declining quickly throughout the world [2,3]. Captive breeding programs have been established for several species of animals, and the increased popularity of captive-bred reptiles can alleviate pressure on wild populations [4,5]. Sex determination has a fundamental role in the context of captive breeding; nevertheless, some species of reptiles have little or no sexual dimorphism [6], and their sex is visually impossible to determine. In every reptile breeding program, sex determination is mandatory for establishing breeding groups in which single-sex subjects do not engage in competitive and aggressive behaviours [7,8,9,10,11,12,13,14,15,16,17,18,19,20,21,22,23,24,25]. Moreover, knowledge of the sex of individual subjects is essential in zoos or private facilities to make reproductive pairs [7,8,9,10,11,12,13,14,15,16,17,18,19,20,21,22,23,24,25,26,27]. The objective of this review is to provide readers with an updated and comprehensive overview of the various sex determination techniques in reptiles as reported and described in the current literature. By presenting the latest advancements and methodologies, this review aims to improve the understanding of diagnostic approaches and their practical application in the most commonly encountered reptile species of veterinary interest, for medical and conservation purposes.

## 2. Material and Methods

The databases Google Scholar, PubMed, and Scopus were searched with the following keywords: “sex identification, sex determination, reptiles, chelonia, squamata, crocodylia, rhinchocephaila”. Only peer-reviewed articles were considered for inclusion. Articles that did not meet this criterion or were deemed irrelevant to the review’s objectives were discarded. 

## 3. Results

Forty-four journal articles and five book chapters were included in this review, having satisfied the criteria described above.

### 3.1. Anatomy of the Reproductive Apparatus of Reptiles

#### 3.1.1. Squamata (Lizards, Snakes, Amphisbenae)

##### Males

In general, the external color of the testes varies within and among the orders and among the species of reptiles, with colors including white, yellow, brown, and black [28]. In many snakes and lizards, the right testis is cranial and to the left [28]. Testes can be either ovoid or elongate, although those of blind snakes in the genus *Leptotyphlops* are described as being multilobed [28,29]. In most species, testis size is influenced by season, whereby the size increases in volume after hibernation or in early summer due to an increase in spermatogenesis [28]. Testes are surrounded by a tunica albuginea. The parenchyma consists of convoluted seminiferous tubules with the interstitium containing fibroblasts, blood vessels, lymphatic vessels, and interstitial cells. Seminiferous tubules are lined with seminiferous epithelium consisting of Sertoli cells and developing germ cells, and they empty into ductuli efferentia [28]. Hence, sperm enter the ductuli epididymides, followed by the ductus epididymis, and finally reach the ductus deferens, where the sperm is conveyed to paired hemipenes [28].

##### Females

The female reproductive tract consists of paired ovaries and pairs of reproductive ducts. Ovaries may be found in the middle to caudal coelomic cavity in lizards [28] and in proximity to the gallbladder in snakes [28]. In snakes, the ovaries are elongated, and similar to lizards, the right ovary is cranial to the left [28]. The ovary contains a hierarchy of follicles at different stages of development, including atretic follicles [28]. Previtellogenic and vitellogenic follicles can be differentiated by size and color [28]. Although previtellogenic follicles are small and whitish, they enlarge as they are recruited for vitellogenesis [28,29,30]. After ovulation, the follicle is received by the oviduct, which travels from the region adjacent to the ovary to the cloaca, where in most species, the paired oviducts open independently [28]. The oviduct is divided into the infundibulum, the uterine tube, the isthmus (aglandular portion), the uterus, and the vagina [28,29,30,31]. In lizards and snakes, the oviducts participate in sperm storage, fertilization, egg transport, eggshell deposition, and egg (or fetus) expulsion [28,31]. In viviparous squamates, the uterine glands are usually sparse and/or inactive throughout most of gestation. Lizards and snakes do not produce an albumen layer in their eggs and thereby lack equivalent glands [28]. Some lizards and snakes possess only one oviduct [28]. In some species of amphisbenae, the right ovary is longer than the left ovary, whereas in other species (*Anops* sp. and *Chirindia* sp.), the left ovary is longer [32].

#### 3.1.2. Chelonia (Turtles, Tortoises, and Terrapins)

##### Males

In chelonians, the testes are globose organs (ovoid and compressed dorsoventrally) with a smooth surface that is yellowish white in color [28,32]. Generally, at the end of hibernation, an increase in size is visible and correlated with active spermatogenesis [28]. In all of the species that go into hibernation, the testicles increase in volume in spring, whereas in the other species, this increase is recorded at the end of the summer period [28]. The testicles are externally lined by the tunica albuginea (serous). Internally, the parenchyma consists of a network of seminiferous tubules and an interstitium containing fibroblasts, blood and lymphatic vessels and interstitial cells. Interstitial cells can vary in shape and number, which is dependent on the reproductive season. The seminiferous tubules are delimited by the seminiferous epithelium, which consists of Sertoli cells and germ cells (spermatogonia near the basement membrane, spermatocytes below the spermatids, and spermatids and spermatozoa near the tubular lumen) [28]. From this site, spermatozoa reach the epididymal ductules up to the vas deferens, which is a lumen delimited by hair cells and simple cells [28]. The vas deferens terminates directly in the vicinity of the phallus, where the sperm reaches the female cloaca during copulation through a ventral sulcus (the urethra is not present) [28].

##### Females

The female reproductive system in chelonians consists of a pair of ovaries and a pair of oviducts. Depending on the species, the ovaries can generally be localized near the caudal third of the coelom, which is proximal to the cranial pole of the kidneys. Mature ovaries contain a hierarchy of follicles at different stages of maturation/atresia [28]. The oviduct (uterus) runs from a region adjacent to the ovary up to the cloaca, where in most species, it travels independently. Histologically, the oviduct wall consists of two layers of smooth muscle that is externally bound by the serosa [28]. Anatomically, as mentioned above for squamata, it is possible to divide the oviduct into five distinct regions: the infundibulum, uterine tube, isthmus, uterus, and vagina. The oviductal lumen is delimited along its path by hair cells and egg white-producing glands. In contrast, the uterine mucosa has glands that secrete calcareous and proteinaceous components for the constitution of eggshells and membranes [28]. The uterus ends in the vagina, which is delimited by cuboidal hair cells. There is no glandular epithelium, and the muscle is well developed in this region. The vagina opens into the cloaca through a common opening or via two distinct outlets.

#### 3.1.3. Crocodylia (Alligators, Caimans, Gharials)

##### Males

Johnston et al. [33] described the reproductive apparatus of the male saltwater crocodile. Paired elongated ovoid testes were shown to be located in a retroperitoneal position and located slightly ventral and cranial to the kidneys, with both the left and right testes being physically separated by a prominent mesocolon. The tunica albuginea of the testis appears to be well vascularized, and the gonads are surrounded by a thick layer of adipose tissue. The ductuli efferentes and ductuli epididymides are not evident on gross dissection. The ductus deferens is covered by a pseudostratified epithelium and opens via a papilla at the proximal end into a single medial groove along the shaft and glans penis, which is known as the sulcus spermaticus [33]. As previously reported by other authors [28,34,35], crocodiles (as chelonians) possess a single penis; it arises from the wall of the proctodeum and is contained in this structure when not erect. The glans penis possesses a hollow cavity that opens towards the tip of the penis, which is not directly connected to the medial groove. The penis of neonatal crocodilians is larger than the clitoris and can be used for sexing of the neonates [28,36].

##### Females

The ovaries are situated cranioventrally to the kidneys and are partially attached to their cranial pole [37]. The ovaries are elongated and clearly distinguishable from testes in immature specimens [37]. In adult animals, all of the follicles mature simultaneously [37]. The oviduct is anatomically similar to that described for squamata; however, in squamata, there is no albumen production by the uterine tube glands, and the uterine tubes of crocodilians are capable of synthetizing and secreting albumen, as in the avian magnum [31]. The uterus is sometimes referred to as a “nonglandular uterus” because the term “vagina” erroneously implies that it receives the male intromittent organ during copulation [28,38]. Crocodilians have a clitoris, which is the homologue of the penis, and it is located on the ventroanterior cloaca [28,36].

#### 3.1.4. Rhynchocephalia (Tuataras)

##### Males

Even though tuataras belong to the Rhynchocephalia order, their reproductive system is consistent with reports in the Squamata [39]. In males, testicles are found in the pleuroperitoneal coelom posterior to the liver. The testes are elongated along the anteroposterior axis and flattened in the dorsoventral axis. The pleuroperitoneum externally covers the testes. Deeper into the body, connective tissue sheaths, such as the tonaca albuginea, cover entire organs [39]. The seminiferous tubules merge with the rete testis tubules, which are contained in a connective sheet that is continuous with the tunica albuginea (the epydidimal sheath) [39]. The ductuli efferentes extend alongside the epydidimus, which is encased in a thin layer of smooth muscle. In proximity to the kidney, the epydidimus transitions into the ductus deferens, which opens in the cloaca through the ampulla ductus deferentis [39].

##### Females

Mature ovaries contain a hierarchy of follicles at different stages of maturation [39]. As in lizards, the oviduct travels from a region adjacent to the ovary and up to the cloaca. Tuatara do not produce an albumen layer in their eggs and lack equivalent glands in the oviduct [28].

## 4. Sexual Dimorphism

**Figure 1:**
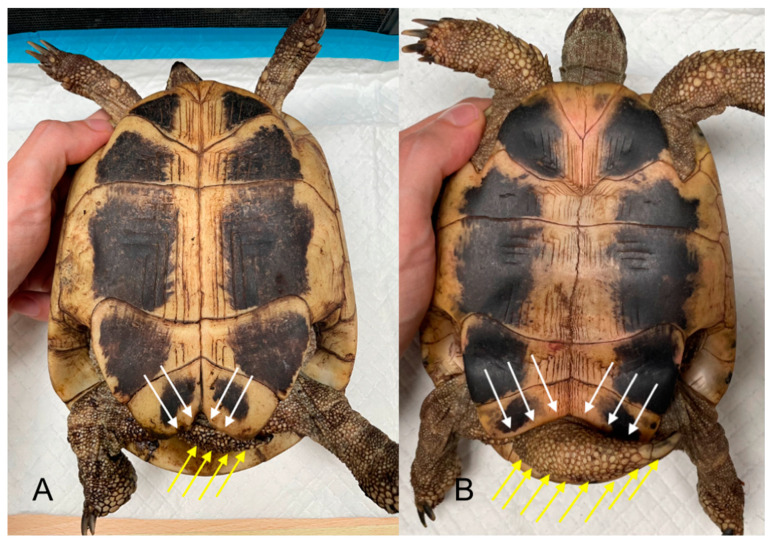
(**A**) Adult female Hermann’s tortoise (*Testudo hermanni*), ventral view. The tail is noticeably smaller (yellow arrows) compared to that of the male (**B**), yellow arrows. The angle formed by the right and left anal scutes is wider in the male (**B**, white arrows) than in the female (**A**, white arrows).

Sexual dimorphism is a condition in which the sexes of the same species exhibit different characteristics, such as different sizes, colors, weights, and other morphological features [8] (Figure 1).

**Figure 2:**
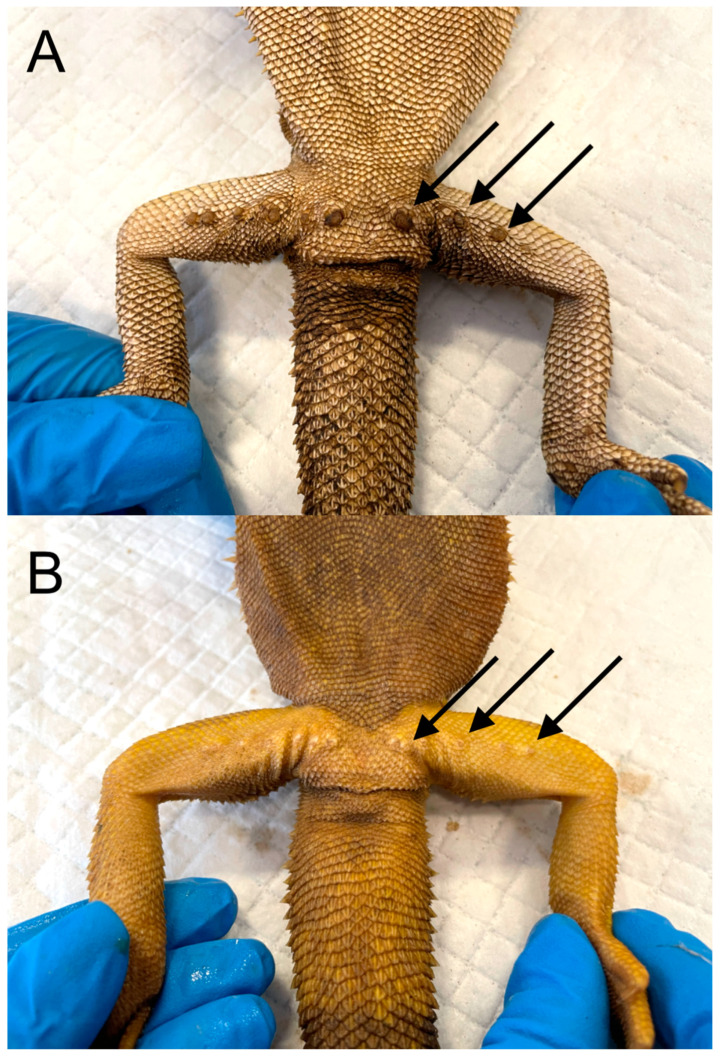
(**A**) An adult, mature male bearded dragon (*Pogona vitticeps*), positioned in dorsal recumbency. The presence of well-developed femoral pores is evident (black arrows). (**B**) An adult, mature female bearded dragon (*Pogona vitticeps*), positioned in dorsal recumbency. Femoral pores are less developed (black arrows).

Phenotypic differences between males and females may be evolutionally related to reproductive success, resulting in partner selection [8]. In some reptiles, the large body size of males may have evolved because their large size promotes success in potential mates and subsequent reproductive success [9]; on the contrary, in some species with variable clutch sizes, mature females are larger than males [9]. This likely confers a reproductive advantage because the number of eggs or offspring increases with body size [10,11]. Animal size is also influenced by hormones, such as testosterone, as reported by Cox and John-Alder [12]. Additionally, the brightness of the skin of animals is influenced by sex hormones in many species [13], which plays important roles in inter- and intraspecific interactions [14], acts as a signal to warn of potential threats (aposematism) [14], and attracts mating partners [15,16,17]. In *Anolis* lizards, the development of a dewlap as an extendable cutaneous fold is found in both males and females, even if males typically have larger and more-colored folds [18]. In *Anolis* sp., the dewlap is an extremely important tool that is used for communicating between individuals, as well as for social interaction, isolation [18], and sex recognition [19]. In some lizards, the femoral glands are situated in the epidermis of the inner thighs and produce a holocrine secretion that travels to the external region through epidermal structures known as “femoral pores” [20]. These structures are present in both sexes but are more developed in males [20,21,22,23] (Figure 2A,B). 

**Figure 3:**
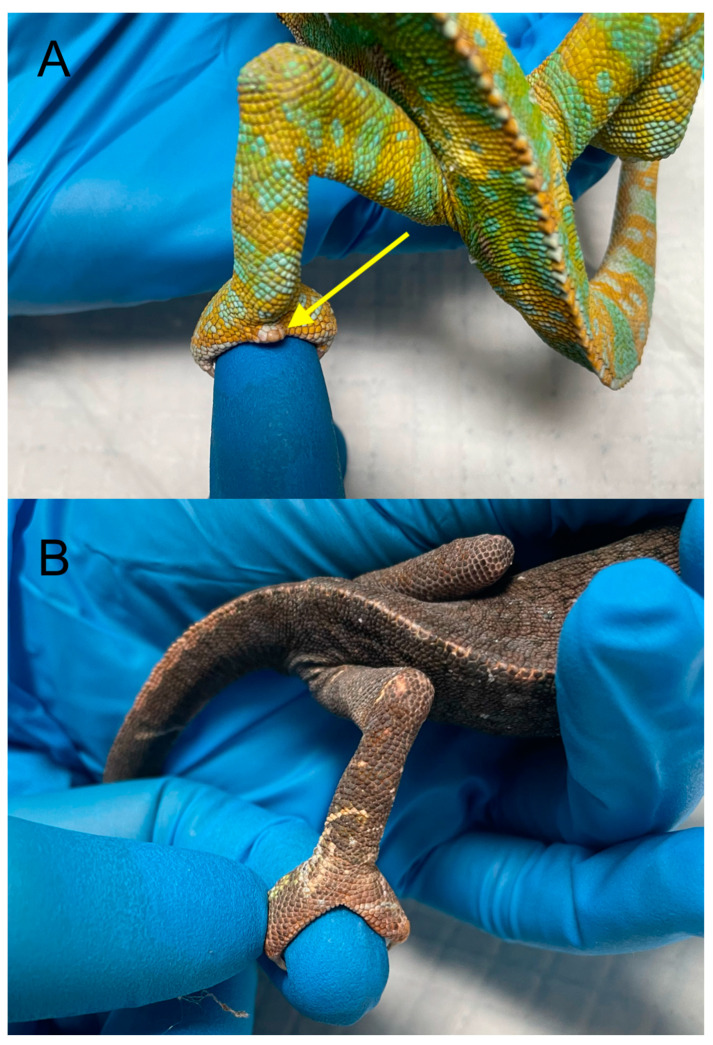
(**A**) subadult male veiled chameleons (*Chamaeleo calyptratus*). A tarsal spur is evident on the lateral aspect of the tarsus (yellow arrow). (**B**) Adult female of the same species. The tarsal spur is absent.

The holocrine excretions of the femoral glands of lacertids primarily function in territory demarcation and/or in mate choice [21,22,23]. The gharials (*Gavialis gangeticus*) are sexually dimorphic in size, and males also possess a sexually selected structure (the ghara), which has an osteological correlate in the presence of a fossa associated with the nares and likely functions as a visual display signal to females [24]. In chameleons, mature males of veiled chameleons (*Chamaeleo calyptratus*) exhibit a well-developed bony helmet consisting of the parietal bones of the skull [25]. Moreover, adult males are much larger than females and have prominent knob-like protuberances (tarsal spurs) on their heels, whereas females lack these structures; this characteristic is easily recognizable starting from the hatchling stage [25] (Figure 3A,B).

**Figure 4:**
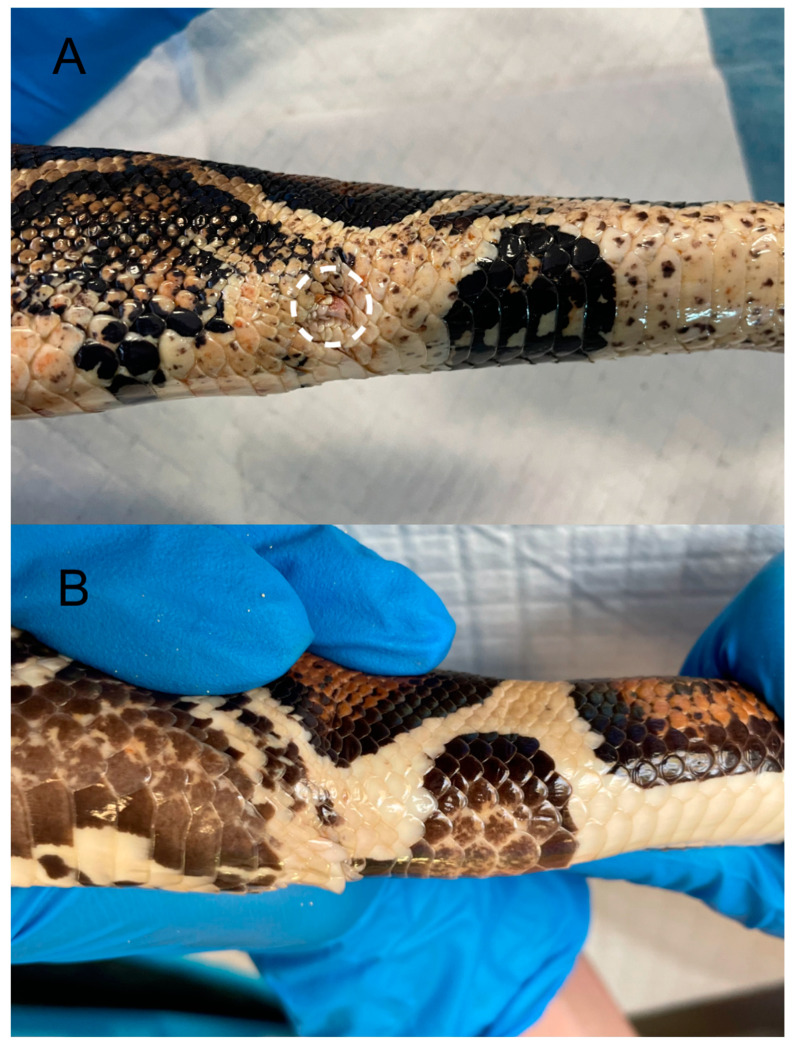
Close-up of the right lateral region dorsal to the cloaca in two specimens of the common boa (*Boa constrictor*). (**A**) Adult male specimen: the dashed circle highlights a well-developed femoral spur. (**B**) Adult female specimen: the spur is not evident.

Another species that demonstrates evident sexual dimorphism is Jackson’s chameleon (*Trioceros jacksonii*) [26]. Only males possess three well-developed bony horns covered by a thick keratin layer (one pair arising from the left and right frontal bones and one single horn arising from the nasal bone) [27]. In specific snake superfamilies within the infraorder Alethinophidia, pelvic spurs are external projections situated near the cloaca. Both males and females possess a pair of spurs, one on either side of the cloaca, with the male’s spurs being longer than those of the female [9] (Figure 4A,B).

## 5. Sex Determination Techniques

### 5.1. Probing

The most common method for determining the sex of adult snakes and certain lizards involves probing of the cloaca with a smooth, blunt instrument. These commercially available probes are of various sizes [40]. This technique aids in identifying the presence of hemipenes in males. The specific utilized tool is not critical, so long as it is long, smooth, and has a blunt tip. Cloacal probing is a relatively simple procedure that can be mastered with practice [40,41]. The animal should be properly restrained, and the probe should be gently inserted into the cloaca and directed in a caudal direction.

**Figure 5:**
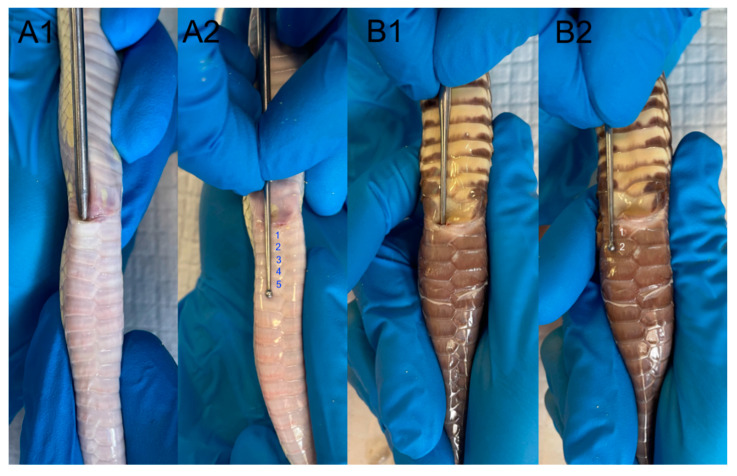
(**A1**) Probing procedure in a male albino California kingsnake (*Lampropeltis getula californiae*). The probe is gently inserted into the hemipenile pocket until resistance to further advancement is encountered. (**A2**) The depth of probe insertion is measured by counting the postcloacal scales. In this case, the probe advanced into the pocket to a depth of five postcloacal scales. This counting method serves as a comparative tool in cases of ambiguous results between different specimens. (**B1**) Probing procedure in a female California kingsnake (*Lampropeltis getula californiae*). Note that in females, the probe advances only to the length of two postcloacal scales (**B2**), compared to males, due to the absence of hemipenes and the reduced length of the hemipenile pockets.

By carefully maneuvering the probe to the side of the midline, it will enter into the inverted hemipenis in males, thus allowing for deeper insertion into the hemipenal sulcus (Figure 5(A1,A2,B1,B2)).

The depth of insertion varies depending on the species [40]. Due to the fact that many lubricants have spermicidal properties, caution should be exercised when probing reptiles that are actively breeding or expected to breed soon. In females, the probe penetrates less deeply due to the presence of a hemipenal homologue (the hemiclitoris), which is shorter and narrower than the hemipenis [6,40,41]. A correctly sized probe should allow for smooth entry into both the male hemipenis and the female hemiclitoris without causing tissue damage. If an excessively sharp or small probe is used or if too much force is applied, the probe may mistakenly penetrate a female in the same manner as in a male by puncturing the hemiclitoris [6,40]. Species with well-developed hemiclitores, such as short-tailed pythons and monitor lizards, are more prone to misidentification via this method. Additionally, in some lizards, this technique may be less effective because of increased musculature in the area, which can hinder probe advancement [41].

### 5.2. Hydrostatic Eversion of Hemipenes

Saline injection for hydrostatic eversion is a more invasive and uncomfortable method, with greater potential for causing injury, thus making it less favorable than other sexing techniques [6,40]. It is recommended that less invasive options be initially explored before considering this approach. The procedure involves the injection of sterile isotonic saline solution into the tail just beyond the expected location of the hemipenes (if the sample is male) [6,40]. Similar to manual eversion, this method involves everting the hemipenes from the base of the tail to identify males [40]. However, unlike manual eversion, it offers a clear anatomical marker for females rather than simply relying on the absence of male traits. If the needle is placed too close to the base of the tail, the fluid may directly enter into the hemipenis rather than behind it, thus rendering the procedure ineffective and increasing the risk of injury [40]. The hydrostatic pressure that is generated in the tail not only forces the hemipenes to evert but also causes the surrounding tissue around the cloaca to swell [6,40]. This swelling partially everts the cloaca, thus allowing for the visualization of the oviductal papillae in females [40].

### 5.3. Popping

“Popping” is a manual technique that is used to determine the sex of juvenile reptiles, particularly snakes. This involves the application of gentle pressure to the base of the tail near the cloaca, which causes the everted hemipenes to protrude in males. This method is often preferred in younger animals, as their reproductive organs are more easily manipulated. In females, no hemipenes are visible, and only slight cloacal eversion may occur [6,40,42,43]. Instead of merely applying pressure, additional anatomical features can sometimes be observed during the “popping” technique. In females, the openings of the scent glands or papillae may be visible, particularly along the lateral edges of the cloaca [40,42]. Although this procedure is effective in skilled hands, improper techniques can injure reproductive structures or the tail. This technique can also be performed with the reptile under anesthesia, which helps to fully relax the retractor muscles and allows for easier eversion of the hemipenes or hemiclitores [40]. Anesthesia can significantly reduce a reptile’s stress and discomfort and decrease the chances of injury due to struggling during the procedure [40].

### 5.4. Ultrasound

**Figure 6:**
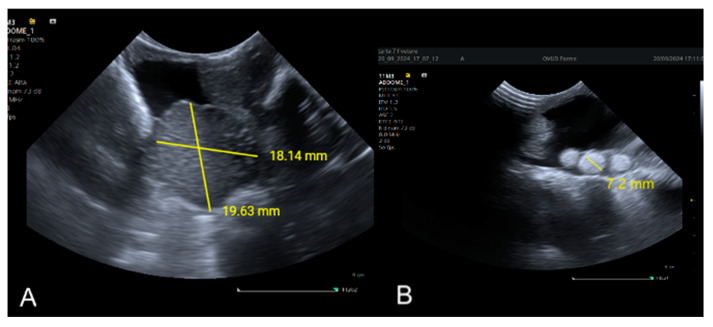
(**A**) Ultrasonographic view showing the right testis (18.14 × 19.63 mm) in a mature male Greek tortoise (*Testudo graeca*) during the breeding season (June). (**B**) Ultrasonographic view showing the right ovary in a mature female Greek tortoise (*Testudo graeca*) after the breeding season (September). Multiple round, hyperechoic, 7 mm diameter early vitellogenic follicles are visible. The ultrasonographic views were obtained from the right prefemoral fossa.

Ultrasound is a valuable tool for noninvasive sex determination in reptiles, particularly in species in which external sexual dimorphism is minimal [40,44]. This technique relies on the visualization of reproductive organs such as ovaries, follicles, or testes. In female reptiles, the presence of ovarian follicles or oviducts can be observed, whereas males are identified by the absence of these structures and the presence of testes, which may appear as elongated structures near the kidneys [6,40,44] (Figure 6A,B).

Ultrasound is particularly useful for monitoring follicular development in gravid females [6,44]. Compared with invasive techniques, this method minimizes stress and the risk of injury [6,40]. A previous study on adult heloderms revealed that ultrasonography was effective in detecting the presence or absence of hemipenes at the base of the tail [44]. However, a more recent study evaluating hemipenal identification across 19 lizards from four different species via various imaging techniques concluded that ultrasound had a relatively low accuracy rate for sex determination, with only 64.3% accuracy being reported [45]. This finding indicates that although it is useful in some contexts, ultrasound may not be the most reliable tool for sexing lizards. Ultrasonographic measurements of hemipenes and hemiclitores were performed on 50 snakes from various families, including *Pythonidae*, *Boidae*, and *Colubridae*. Statistically significant correlations were identified between hemipene dimensions and certain morphometric parameters, whereas no similar correlation was observed for hemiclitores dimensions. The procedure can be conducted without the need for anesthesia or sedation and relies on manual restraint. These findings support ultrasonography as being a reliable, noninvasive technique for sex determination in snakes, thus highlighting its sensitivity and safety in comparison with more invasive methods [46]. In blue tongue skinks (*Tiliqua* sp.), the use of ultrasound for sex determination is often ineffective because of their thick scales, which hinder the penetration of sound waves [41].

### 5.5. CT Scan

**Figure 7:**
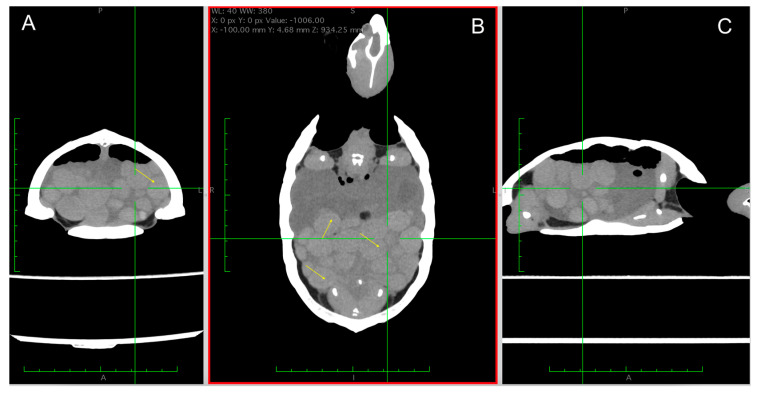
CT scans of a female common musk turtle (*Sternotherus odoratus*) affected by preovulatory stasis. (**A**) Transverse plane, (**B**) coronal plane, and (**C**) sagittal plane, showing numerous altered follicles filling the caudal coelom, with (hypodense areas yellow arrows) alternating with hyperdense areas of the yolk.

Many studies have investigated the use of CT scans as tools for sex identification in reptiles [40,45,47,48]. Even though these advanced imaging modalities are not available in most private clinical practices, CT is becoming accessible in many veterinary hospitals. One advantage of CT over radiography is its ability to individually evaluate each ovarian follicle and oviductal egg. In one report on imaging of the reproductive tract of reptiles, CT (compared with other imaging modalities such as radiology and ultrasonography) was the most informative technique for accessing the chelonian reproductive tract. Moreover, CT is useful for identifying advanced changes in follicular stasis (such as horizontal levelling of the follicle’s yolk) [40,47] (Figure 7A–C).

This alteration can also be detected in retained oviductal eggs; moreover, with more chronic retention, a gas cap often forms within the egg (along with gas pockets formed around the egg) [47]. In a study by Kircher et al. [48], volumetric data obtained via noninvasive imaging modalities, such as microcomputed tomography (microCT) and optical coherence tomography (OCT), provided unprecedented structural insights into the female reproductive tract of the brown anole (*Anolis sagrei*). This study highlighted how these technologies offer significant advantages over traditional histological methods; although these histological methods are effective for characterizing the cellular composition, they fail to preserve the three-dimensional (3D) relationships between organs and tissues [48].

### 5.6. Radiography

**Figure 8:**
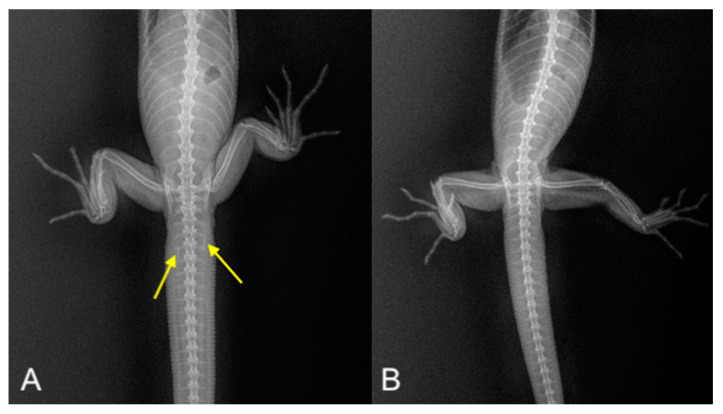
On the left (**A**), radiograph of a mature male black-headed monitor (*Varanus tristis*), showing evident mineralized hemibacula (yellow arrows). On the right (**B**), a mature female of the same species; hemibacula are absent. Courtesy of Dr. Clément Paillusseau.

Radiography in reptile sex determination involves X-ray images to detect sexual structures such as hemibacula, which are cartilaginous structures found in the hemipenes of some male reptiles (such as some species of monitors). When they reach sexual maturity, these structures become mineralized and are visible on radiographs, thus allowing for differentiation between males (with hemibacula) and females (without hemibacula) and aiding in accurate sex identification [40] (Figure 8A,B).

### 5.7. Contrast Radiography

Contrast radiography in reptile sex determination involves the injection of an iodinated contrast medium into the hemipenal or hemiclitoral sac to visualize internal reproductive structures. The contrast agent highlights the differences between male hemipenes, which appear larger and spiral-shaped, and female hemiclitores, which are thinner and straighter. This technique enhances the clarity of radiographic images for accurate sex identification [6,40,41,45]. In a study by Vetere et al. [6,41,49], contrast radiography was used to determine the sex of young Sierra Nevada lizards (*Timon nevadensis*), with promising results being achieved. The initial attempt at sexing was performed via cloacal probing, which is a technique that is commonly used for reptiles. Eleven males and eight females were identified; however, the results were inconclusive for four individuals. To improve accuracy, contrast radiography was subsequently performed, thus revealing 14 males and 9 females. After 8 months, ultrasound rechecks confirmed sex determination for 15 of the 14 previously identified males, and all 9 females were correctly identified. This study suggests that contrast radiography may have greater sensitivity and accuracy in sex determination than cloacal probing, thus making it a valuable, less invasive tool for the sexing of young lizards [6]. Another study compared the accuracy of two different techniques (contrast radiography and cystoscopy). For forty-two Indonesian blue-tongued skinks (*Tiliqua gigas*), cystoscopy was the primary method that was used for sex identification, and it successfully visualized the gonads in 98% of the subjects. However, the same study reported that contrast radiography showed limited accuracy, with a sensitivity of 69.6% and a specificity of 75% for identifying female skinks. Although the cystoscopy technique was highly effective, contrast radiography in this species yielded mixed results, thus indicating that its usefulness may vary between species and individual cases. Another study by Vetere et al. [41] explored the use of a noninvasive radiographic technique for sex identification in 42 eastern blue-tongue skinks (*Tiliqua scincoides scincoides*). The method involved the introduction of a radio-opaque solution (1 mL of iohexol) into the hemipenal or hemiclitoral sac via a syringe and catheter. Contrast material was injected as the catheter was gradually withdrawn, and the skinks were then radiographed. The resulting images demonstrated distinct, repeatable differences between hemipenes and hemiclitores. In the dorsoventral view, the hemipenes appeared to be wider with a spiraling outline, whereas the hemiclitores were thinner and had a sharper, angled outline. In the horizontal view, the hemipenes exhibited a spiral shape, whereas the hemiclitores were straight and horizontal. This technique achieved 100% accuracy in correlating with breeding records and coelioscopic sexing, thus making it an effective tool for skinks of 15 cm snout-to-vent length (SVL) or larger. In contrast, a study involving 23 Gila monsters (*Heloderma suspectum*) evaluated the effectiveness of hemipenis contrast radiography and computed tomography (CT) for sex determination, with this study comparing these methods with coelomic ultrasound. The results were less promising for contrast radiography, which identified hemipenes in only 1 out of 15 males (7%). However, CT imaging improved this detection rate to 60% (9/15 males). In females, contrast radiography and CT successfully outlined the cloacal rim in 100% of the animals (8/8 females). Despite these findings, the study concluded that both contrast radiography and CT were unreliable for sex determination in Gila monsters [48]. In another study, hemipenile contrast radiography was evaluated for sex determination in 11 captive adult beaded lizards (*Heloderma alvarezi* and *Heloderma horridum*). The results from contrast radiography were compared with those from coelomic ultrasound, with both methods identifying two females and nine males. The study demonstrated a perfect agreement (Kappa concordance of 1) between the two techniques, thus indicating that contrast radiography is a reliable method for sex determination in beaded lizards. This technique successfully identified hemipenes, thus suggesting that it could be a useful diagnostic tool for species with limited sexual dimorphism [50].

### 5.8. Endoscopy

Endoscopy is a minimally invasive technique used for sex determination in reptiles because it allows for the direct visualization of internal reproductive organs. This method is highly accurate, especially in species with little to no external sexual dimorphism. Generally, a rigid endoscope with a 2.7 mm telescope and a 4.8 mm sheath system equipped with an operative sheath is used. Endoscopy can be performed either by directly visualizing the gonads through the coelom (coelioscopy) or through the urinary bladder wall after the endoscope is inserted into the urinary bladder through the urethral opening.

### 5.9. Coelioscopy

**Figure 9:**
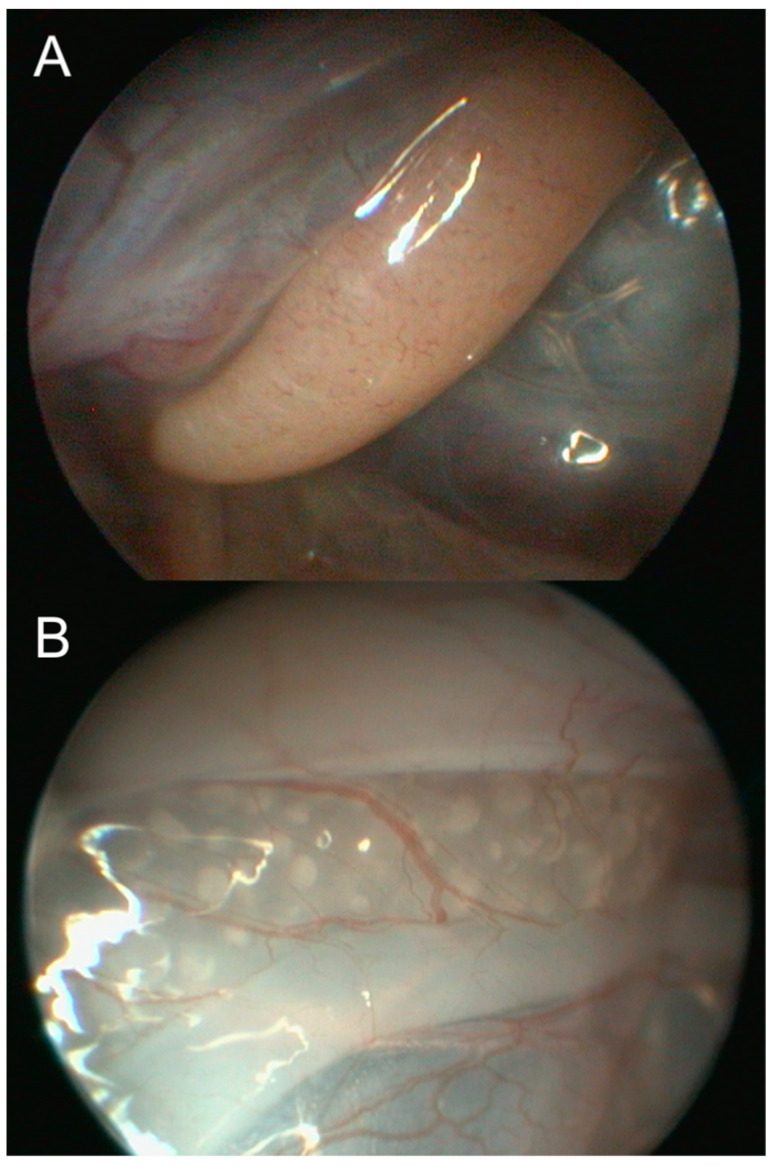
(**A**) Coeloscopic image of an immature testis in an immature male Indian star tortoise (*Geochelone elegans*). The testis appears as a smooth, pink to orange, elongated structure. (**B**) Coeloscopic image of an immature ovary in an immature female Indian star tortoise (*Geochelone elegans*). The ovary appears as a yellowish, elongated structure composed of small clusters of clear, fluid-filled follicles.

Celioscopic sex identification is a procedure that involves the direct identification of a reptile’s gonads using a tool such as a rigid endoscope [40]. In chelonians, the procedure is performed under general anesthesia or sedation [49] and involves making a small incision at the right or left flank fossa to allow the insertion of the endoscope sheath. The iliacus and transverse muscles, as well as the coelomic membrane, are incised to provide access to the coelom. The endoscope is directed caudodorsally within the coelom until the gonads are visualized and identified [40,49]. Testes appear as smooth, ovoid to elongated structures (Figure 9A), while immature or inactive ovaries are small clusters of clear, fluid-filled follicles (Figure 9B).

In lizards, a small incision (2–4 mm) is made at the chosen entry site after aseptic preparation. The skin, underlying musculature, and coelomic membrane are carefully incised or bluntly dissected to allow the insertion of an endoscope sheath and obturator into the coelomic cavity [51]. Insufflation with CO₂ at pressures between 0.4–0.7 KPa (3–5 mmHg) can be performed to create pneumocoelom, providing a clear view of the coelomic structures [51]. The gonads, located along the dorsal midline in the mid-coelom, are visualized to determine sex. Testes appear as smooth, ovoid structures, while immature or inactive ovaries are small clusters of clear, fluid-filled follicles [51]. Active gonads may be significantly enlarged during reproductive seasons. This technique is particularly advantageous for species with no external sexual dimorphism or when non-invasive methods fail to provide definitive results. It offers a high degree of accuracy while minimizing trauma and recovery time [51]. Coelioscopy in snakes presents greater challenges compared to lizards, primarily due to their elongated body structure, which prevents comprehensive examination of all organs from a single entry point. Furthermore, the presence of diffuse fat bodies and complex fascial planes complicates both insufflation and instrument navigation [51]. However, since good results can often be achieved with less invasive techniques such as ultrasound and probing, coelioscopy is rarely used [6,40,42,43,44]. In crocodilians, dorsal recumbency is preferred for a ventral paramedian entry, as their osteoderms make the paralumbar approach challenging [51]. In American alligators (Alligator mississippiensis), a minimally invasive laparoscopic surgical technique has been successfully employed to examine and describe the gonads of live females as part of reproductive assessments in the context of conservation medicine and biology [52].

### 5.10. Cystoscopy

**Figure 10:**
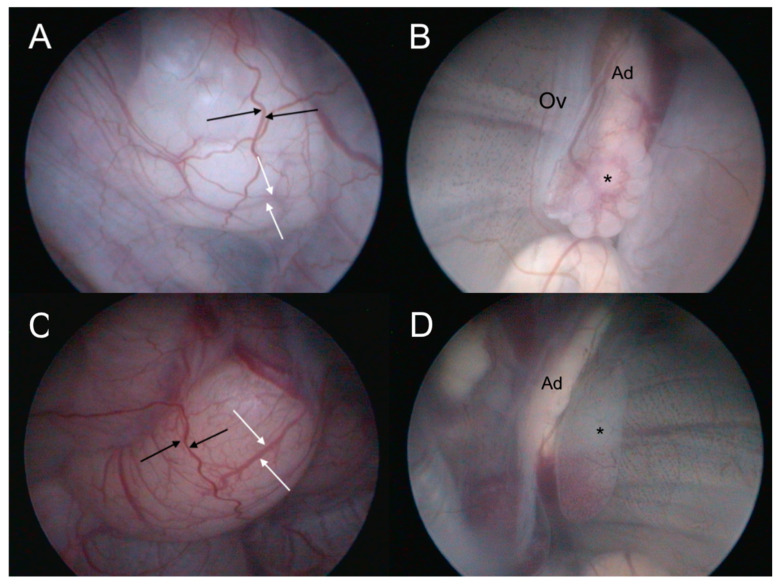
Cystoscopic appearance of gonads in Indonesian blue-tongued skink (*Tiliqua gigas*). (**A**) Ovary of a subadult, sexually immature specimen. Black arrows indicate blood vessels associated with the bladder, while white arrows indicate ovarian vessels. (**B**) Ovary of a sexually immature specimen weighing only 28 g. Several ivory-colored follicles of equal size are visible (asterisk). Laterally to the adrenal gland (Ad), the oviduct can be observed as a flattened, whitish structure running parallel to the ovary. (**C**) Testis of a sexually mature adult specimen. Black arrows indicate blood vessels associated with the bladder, while white arrows indicate testicular vessels. (**D**) Testis of an immature specimen weighing 26 g. A pale red, immature testis is marked by an asterisk. Ad: adrenal gland.

This technique cannot be performed on snakes, crocodiles, or any species that lack a urinary bladder (Table 1). Cystoscopic sex identification in reptiles is performed through different steps; specifically, the urethra is endoscopically identified in the urodeum and accessed, thus reaching the urinary bladder [41]. The coelom is inspected through the transparency of the bladder until the gonads are detected. A 30° viewing angle is advantageous once the bladder is accessed, as it allows for optimal visualization of the gonads, which are positioned dorsally and slightly caudally relative to the center of the urinary bladder [53,54]. To date, cystoscopic sex identification has been performed in various reptilian species via a 2.7 mm, 30° rigid endoscope with reliable results [41,53,54]. In small chelonians and small skinks, a rigid endoscope is well suited for sex identification because of the extreme shortness of the urethra and its alignment along an imaginary straight line that is parallel to the long axis of the animal. This anatomical configuration minimizes the need for extensive maneuvering of the endoscope, thus making lateral adjustments rarely necessary [41,53]. Warm fluid infusion allows for the opening of the urethra and distension of the bladder. Once the bladder is distended, it is possible to observe transparent gonads located in the caudodorsal coelom [41,53,54]. In posthatchling Hermann’s tortoises, ovaries are described as being elongated, white-to-translucent, and slightly convoluted organs with whitish follicles, whereas testes are oval-to-round, tan–yellow in color, and have a smooth surface with a tight network of blood vessels [53]. In that study, although traumatic bladder ruptures were described [52], no complications were reported. In skinks, gonad appearance changes with body size [53] (Figure 10A–D). Smaller males typically have pale red testicles with a fine vascular texture, which turn white, beige, or pale yellow as body size increases [53] (Figure 10D). In the study reported by Vetere and colleagues, cystoscopic examination proved to be more accurate than contrast radiography.

### 5.11. Cloacoscopy

**Figure 11:**
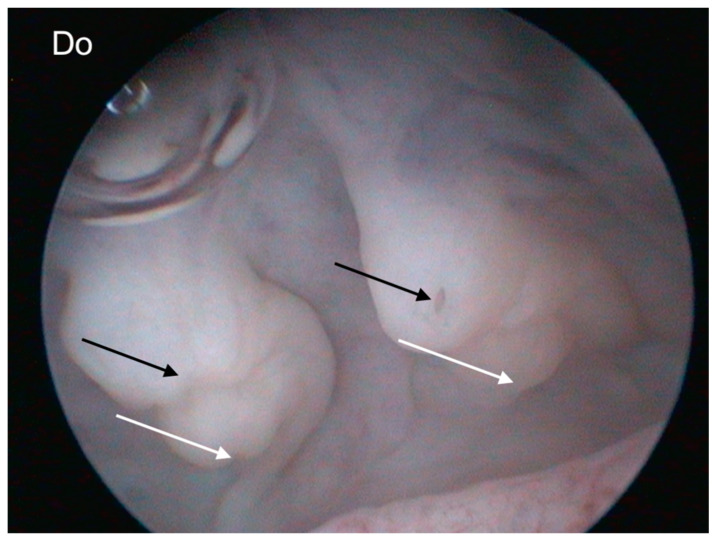
Cloacoscopic appearance of the urodeum in an adult female blue tongue skink (*Tiliqua gigas*). Four papillae are visible dorsally at the level of the urodeum. The two cranial papillae represent the outlets of the oviducts (white arrows), while the two caudal papillae (black arrows) correspond to the openings of the ureters. Do: dorsal.

Endoscopic examination of the cloaca (cloacoscopy) for sex identification has also been described in reptiles. The technique is based on the identification of four papillae in the urodeum of females: two cranial papillae corresponding to the openings of the oviducts and two caudal papillae corresponding to the openings of the ureters (ureteral papillae). In mature males, only the two ureteral papillae are present [49,55,56,57] (Figure 11).

Cloacoscopy for early sex identification was used in one-year-old *Varanus cumingi* and *Varanus macraei.* In this study, sex was accurately determined by observing the urodeum (which is blind-ended in males) and two ostia (oviduct outlets) in females [56]. Via cloacoscopic examination in sexually mature chelonians, it is possible to identify the phallus located on the floor of the urodeum in males and the clitoris in females [57]. However, in some cases, hormonal imbalances can cause females to develop a pseudophallus with features resembling those of a true phallus, such as a groove, glans, and corpus cavernosum. This makes accurate sex determination more difficult and increases the risks of gender misidentification [58]. In thirteen healthy adult skinks, including six Gidgee spiny-tailed skinks (*Egernia stokesii*), three Hosmer’s spiny-tailed skinks (*Egernia hosmeri*), and four tree crevice skinks (*Egernia striolata*), cloacoscopy successfully identified the sex of the animals. Specifically, male skinks were identified by the presence of urethral papillae and a single horizontal septum in the urodeum, whereas females had two septa, with a central-dorsal fold dividing the urodeum into two pouches [59].

### 5.12. Genotypic Sex Determination (GSD)

Squamate reptiles exhibit significant taxonomic diversity, which is mirrored in their karyotypes. This diversity is evident in variations in chromosome structure, number, and morphology. Additionally, squamates display the emergence of both simple and complex sex chromosome systems, with either male or female heterogametic patterns [60]. Unlike those of other vertebrates, the karyotypes of squamate reptiles typically lack distinct banding patterns. Most of the heterochromatin in these species is concentrated in telomeric or centromeric regions or, when present, on differentiated heterogametic sex chromosomes (Y/W) and B chromosomes [60]. In contrast to mammals, where males always exhibit heterogametic sex, reptiles exhibit variations in heterogametic sex across different species and genera. This variability indicates that a method that is effective for one species may not necessarily apply to others. In many species, the development of testes or ovaries is determined by an individual’s genotype at specific loci, which is a process known as genotypic sex determination (GSD). In species with GSD, distinct sex chromosomes may be present (e.g., ZZ males and ZW females or XY males and XX females), although this is not always the case [60]. Interestingly, snakes exhibit the full spectrum of sex chromosome differentiation. In basal species such as those in the Boidae family, sex chromosomes exhibit minimal differentiation, whereas in more evolved species, such as viperid snakes, the sex chromosomes are fully differentiated [60,61]. Colubridae snakes display an intermediate level of differentiation, thus making snakes an ideal group for comparative studies on the evolution of sex chromosomes [61,62]. Currently, molecular techniques, such as PCR, are primarily valuable for conservation efforts, especially for endangered species. In Komodo dragons, PCR analysis has been further explored and has consistently identified a male-specific longer DNA fragment, thus proving to be useful for molecular sex determination [61]. Male heterogamy has been observed in the lizard *Sceloporus poinsettia*, wherein the diploid number differs between the sexes. Males have a diploid number of 2n = 31, whereas females have 2n = 32. This system is referred to as X1X2Y in males and X1X1X2X2 in females, thus indicating a complex sex chromosome arrangement [62]. In certain lizards of the genus *Lacerta*, females differ from males in their diploid chromosome number, thus reflecting a form of sex chromosome differentiation. This distinction suggests a system of sex determination in which chromosome numbers between the sexes are not equal [63]. Genotypic sex determination by karyotyping remains a complex technique that is primarily used for research purposes rather than in clinical practice [6,64]. Additionally, it is currently applicable to only a small fraction of the many reptile species that are available in the pet trade.

## 6. Conclusions

Accurate sex determination in reptiles is a fundamental aspect of their management in both veterinary medicine and conservation efforts. This review provides an updated overview of the current methodologies for sex determination, highlighting the strengths, limitations, and applicability of each technique across different reptilian taxa. By synthesizing the available literature, this work aims to aid veterinarians, researchers, and conservationists in selecting the most appropriate method based on the species, context, and resources available. The absence of universal external sexual dimorphism in many reptilian species necessitates the use of diverse approaches, ranging from simple probing and manual eversion techniques to advanced imaging modalities such as ultrasound, computed tomography, and endoscopy. Each of these methods has its unique advantages and challenges, emphasizing the need for a tailored approach to individual cases. For instance, while imaging techniques provide non-invasive and highly accurate alternatives, they may not always be accessible due to their cost and the need for specialized equipment. Emerging molecular tools, including genotypic sex determination, present promising avenues for non-invasive and highly reliable sexing methods, particularly in endangered species where invasive procedures may not be feasible. However, these technologies are still in the developmental phase and are not yet widely available for routine clinical or field use. Further research and technological advancements are needed to make these tools more accessible and cost-effective. The integration of accurate sex determination techniques is essential for the success of breeding programs, the prevention of aggression among individuals, and the establishment of genetically diverse populations. Moreover, these methods are critical in understanding and mitigating the impacts of environmental changes on reptilian populations. As new techniques are developed, their validation and application will be crucial in advancing the knowledge and conservation of these unique and ecologically significant animals. By fostering collaboration among veterinarians, researchers, and conservationists, we can ensure the effective preservation and management of reptilian biodiversity in the face of global challenges.

## Figures and Tables

**Table 1:** Overview of taxa categorized by the presence, rudimentary development, or absence of urinary bladders in reptiles [40].

Taxa With Well-Developed Urinary Bladders	Taxa with Rudimentary Urinary Bladders	Taxa Without Urinary Bladders
Chelonians	Lizards	Snakes
Rhynchocephalia	Agamidae	Crocodilians
Lizards	Gekkonidae: Coleonyx	Lizards
Xantusiidae	Iguanidae: *Callisaurus*, *Cophosaurus*, *Crotaphytus*, *Sceloporus*, *Urosaurus*	Anguidae, Chamaeleonidae, Gekkonidae (*Gekko, Phelsuma, Hemidactylus*), Helodermatidae, Iguanidae (*Anolis, Iguana*), Lacertidae, Scincidae, Teiidae (except *Cnemidophorus tigris*), Varanidae (except *Varanus marmoratus*), Pygopodidae, Iguanidae

## Data Availability

Not applicable.
